# Circ_0005736 promotes tenogenic differentiation of tendon-derived stem cells through the miR-636/MAPK1 axis

**DOI:** 10.1186/s13018-023-04115-7

**Published:** 2023-09-05

**Authors:** Guangzhao Yang, Fei Chen, Chunyan Zhang, Chenlin Gu

**Affiliations:** 1 Department of Sports, Communication University of China, Nanjing, No.26, Pengshan Road, Jiangning District, Nanjing, 211172 China; 2 Faculty of Cultural Management, Communication University of China, Nanjing, Nanjing, China

**Keywords:** circ_0005736, miR-636, MAPK1, Tenogenic differentiation, TDSC, Physical activity

## Abstract

**Background:**

Tendon-derived stem cells (TDSCs) are one of stem cells characterized by greater clonogenicity, tenogenesis, and proliferation capacity. Circ_0005736 has been shown to be decreased in Rotator cuff tendinopathy. Here, we investigated the function and relationship of circ_0005736 in TDSC tenogenic differentiation.

**Methods:**

Transforming growth factor β1 (TGF-β1) was used to induce the tenogenic differentiation in TDSC. Cell proliferation, invasion and migration were evaluated by Cell Counting Kit-8, 5-Ethynyl-2′-deoxyuridine, transwell, and wound healing assays, respectively. The detection of the levels of genes and proteins was performed by qRT-PCR and Western blot. The binding between miR-636 and circ_0005736 or MAPK1 (Mitogen-Activated Protein Kinase 1) was verified using dual-luciferase reporter assay and RIP assays.

**Results:**

TGF-β1 induced tenogenic differentiation by enhancing the production of tendon-specific markers and TDSC proliferation, invasion and migration. TGF-β1 treatment promoted circ_0005736 expression, knockdown of circ_0005736 abolished TGF-β1-induced tenogenic differentiation in TDSCs. Mechanistically, circ_0005736 acted as a sponge for miR-636 to up-regulate the expression of MAPK1, which was confirmed to be a target of miR-636 in TDSCs. Further rescue assays showed that inhibition of miR-636 could rescue circ_0005736 knockdown-induced suppression on TGF-β1-caused tenogenic differentiation in TDSCs. Moreover, forced expression of miR-636 abolished TGF-β1-caused tenogenic differentiation in TDSCs, which was rescued by MAPK1 up-regulation.

**Conclusion:**

Circ_0005736 enhanced TGF-β1-induced tenogenic differentiation in TDSCs via increasing the production of tendon-specific markers and TDSC proliferation, invasion and migration through miR-636/MAPK1 axis.

## Introduction

Rotator cuff tendinopathy (RCT) is a common musculoskeletal disorder featured by pain and weakness during elevation and external rotation and is one of the common causes of shoulder pain in physical activity and in the workplace [1, 2]. Currently, clinical treatments for tendon injuries range from physical therapy or surgery, or nonsteroidal anti-inflammatory drugs or corticosteroid injections, which are limited to pain or symptom relief [3, 4]. However, about 40% of RCT patients do not respond to conservative treatment, and over 50% of patients report long-term recurrence and persistent pain [2, 5]. Tendon-derived stem cells (TDSCs) are one of stem cells with greater clonogenicity, tenogenesis, and proliferation capacity relative to tendon cells and can differentiate into tendon cells and induce the formation of the tendon extracellular matrix protein (ECM), thereby healing tendon injury [6, 7]. Therefore, a better understanding on the tenogenic differentiation in TDSCs may benefit for developing a new effective cell-based therapies for RCT patients.

As one of types of noncoding RNAs possessing the covalently closed loop, circular RNAs (circRNAs) are high stability relative to linear RNAs and have been identified to have regulatory functions in diverse physiological functions [8–10]. Besides that, increasing proofs have proved that circRNAs can function as potential therapeutic targets due to their high biostability and pharmaceutical stability [11, 12]. In tendon injury, Yu et al*.* manifested that circRNA-Ep400 transfer via exosomes (Exo) from M2 macrophage accelerated the fibrosis of peritendinous after tendon injury [13]. Han et al*.* found that decrease of circPVT1 induced tendon stem/progenitor cell (TSPCs) senescence progression, and impaired TSPC tenogenic differentiation, self-renewal and migration [14]. In addition, Ge’s team identified 94 differentially expressed circRNAs in RCT and suggested that circRNAs might have roles in RCT via competing endogenous RNA (ceRNA) network, moreover, they found that circ_0005736 in RCT was decreased [15]. Circ_0005736 is originated from RNF24 gene in chr20: 3925823–3955047, given the down-regulation of circ_0005736 in RCT, we the investigated whether circ_0005736 could affect tenogenic differentiation in TDSCs to regulate RCT recovery.

Here, this study investigated the role of circ_0005736 in TDSC tenogenic differentiation to uncover the potential effects of it on RCT. Besides that, circRNAs have been recognized that can function as ceRNAs to affect the level of downstream genes by sequestering microRNAs (miRNAs) [16, 17], and thus, the ceRNA networks of circ_0005736 in TDSCs were also identified to clarify the regulatory mechanism of circ_0006640 in RCT.

## Material and methods

### Cell culture and treatment

Human tendon-derived stem cells (TDSCs) (Chinese Academy of Sciences, Shanghai, China) were grown in low-glucose DMEM plus 10% fetal bovine serum (FBS), and 1% penicillin–streptomycin (all from Procell, Wuhan, China) at 37 °C in a 5% CO_2_ incubator. To induce tenogenic differentiation, TDSCs were treated with 5 ng/mL transforming growth factor β1 (TGF-β1) in low-glucose DMEM for 10 d. The medium was changed every 2 d. Cells at passages 4–6 were collected for subsequent analysis.

### Western blotting

Total proteins were extracted using pre-cooled RIPA lysis buffer (Beyotime, Beijing, China), then separated by 10% SDS-PAGE gels, followed by shifting onto PVDF membranes. Then, the membranes were probed with primary antibodies at 4 °C for 12 h and then HRP-conjugated antibodies for 2 h at 37 °C. The primary antibodies included Scleraxis (SCX) (1:2000, ab58655), mohawk homeobox (MKX) (1:2000, ab236400), Collagen1A1 (COL1A1) (1:1000, ab34710), Fibromodulin (FMOD) (1:1000, ab267465) and GAPDH (1:100, ab181602) and were obtained from Abcam (Cambridge, MA, USA). The ECL procedure (Merck Millipore) was adopted for proteins observation, and the gray value was evaluated using ImageJ soft (National Institutes of Health, Bethesda, MD, USA).

### Cell counting Kit-8 (CCK-8) assay

TDSCs were seeded into 96-well plates at a density of 1 × 104 cells/well overnight; then, each well was added with 10 μL CCK-8 solution (Beyotime) and incubated for 2 h. Subsequently, the absorbance was assessed at 450 nm to assess cell viability.

### 5-Ethynyl-2′-deoxyuridine (EdU) assay

In brief, TDSCs were incubated with 0.5 mM EdU (Abcam) for 24 h in a 96-well plate, fixed by 4% formaldehyde, and then treated with 0.3% Triton X-100 (Beyotime), followed by reacting with click reaction solution for 30 min. The diamidine phenylindole (DAPI) was used to dye the nuclei. Lastly, the images of EdU-positive cells were captured and cell number was calculated.

### Transwell invasion assay

The upper chamber of 24-well Transwell plates (8 um pore size; Merck Millipore, Billerica, MA, USA) was pre-coated with Matrigel™ (Costar, Corning, NY, USA). The assigned TDSCs were plated into the upper chambers, and 500 µL complete medium supplemented with 10% FBS was plated into the lower chambers. Invaded cells on the bottom of the membrane were fixed in methanol after 24 h, and dyed with 0.1% crystal violet (Beyotime), stained cells were then visualized and counted under a microscope.

### Wound healing assay

TDSCs with complete growth medium were planted into a 24-well plate. When cells grew to a fully confluent monolayer, a wound was generated using a 1-mL sterile tip (time 0). Cells were the washed with PBS for twice to remove detached cells and then incubated with in serum-free medium. 24 h later, wound gap distance was recorded and photographed (time 24 h), and cell migration was assessed.

### Quantitative real-time PCR (qRT-PCR)

Total RNAs were extracted adopting TRIzol reagent (Invitrogen, Carlsbad, CA, USA), and then reversed-transcribed into cDNAs using Prime Script RT Reagent Kit (Takara, Dalian, China) and Random or Oligo (dT)_18_ primers. Then, qRT-PCR analysis was performed using SYBR Premix DimerEraser (Takara) to measure the levels of circ_0005736 and MAPK1. For miR-636 detection, Qiagen One-Step RT-PCR kit and SYBR-Green Master Mix were used for reverse transcription and amplification reaction, respectively. Primers are listed in Table 1.

**Table 1:** Primers for qRT-PCR

Name		Primers for qRT-PCR (5′-3′)
hsa_circ_0005736	Forward	TTTACATGAGAACTTCCAGCA
	Reverse	TGGGAAATCCGAGCTCATGG
MAPK1	Forward	CCCGTCTTGGCTTATCCACT
	Reverse	TACATACTGCCGCAGGTCAC
miR-636	Forward	GTATGAGUGUGCUUGCUCGUCCCGC
	Reverse	TCGTGGAGTCGGCAATTCA
GAPDH	Forward	AGAAGGCTGGGGCTCATTTG
	Reverse	AGGGGCCATCCACAGTCTTC
U6	Forward	CTTCGGCAGCACATATACT
	Reverse	AAAATATGGAACGCTTCACG

### RNase R and actinomycin D treatment

Isolated RNAs (about 3 µg) were treated with 5 U/μg RNase R or Mock at indoor temperature for 20 min, then, the resulting RNA was gathered and levels of circular and linear RNAs were detected by qRT-PCR analysis.

TDSCs were incubated with 2 μg/mL Actinomycin D for indicated times; then, RNAs were extracted and subjected to qRT-PCR analysis.

### Cell transfection

The circ_0005736-specific small interference RNAs (siRNAs) and the nontarget siRNAs (si-NC), pcDNA3.1-MAPK1 overexpression plasmids (MAPK1) and the empty plasmids (pcDNA), as well as miR-636 mimics or inhibitor (miR-636 or anti-miR-636) and the contrasts (miR-NC or anti-miR-NC), were constructed by Genema (Shanghai, China). Then, Lipofectamine 2000 (Invitrogen) was applied for transient transfection. Following 48 h transfection, cells were treated with 5 ng/mL TGF-β1 for subsequent analysis.

### Dual-luciferase reporter assay

The fragments of miR-636 with circ_0005736 and MAPK1 binding sites were inserted into the psiCHECK-2 vector (Promega, Beijing, China) to establish wild-type (WT) vectors (WT-circ_0005736/MAPK1 3’UTR). Then, the mutated seed sequences were amplified and the mutation (MUT) vectors (MUT-circ_0005736/MAPK1 3’UTR) were established. Then, 200 ng recombinant vectors and 50 nM miR-636 or miR-NC were transfected into TDSCs, and the luciferase activity was measured 48 h later.

### RNA immunoprecipitation (RIP) assay

TDSCs were lysed in RIP lysis buffer and incubated with A/G magnetic beads (Millipore) and anti-Ago2 antibody or IgG antibody (Abcam). Following proteinase K incubation, beads-binding complexes were purified, and RNAs were detected by qRT-PCR assay.

### Statistical analysis

The data were manifested as mean ± standard deviation (SD). Group comparison was conducted using Student’s* t* test, or Mann–Whitney (two groups), or ANOVA followed by Tukey’s post-test. *P* < 0.05 suggested statistically significant.

## Results

### TGF-β1 treatment induces tenogenic differentiation in TDSCs

**Fig. 1:**
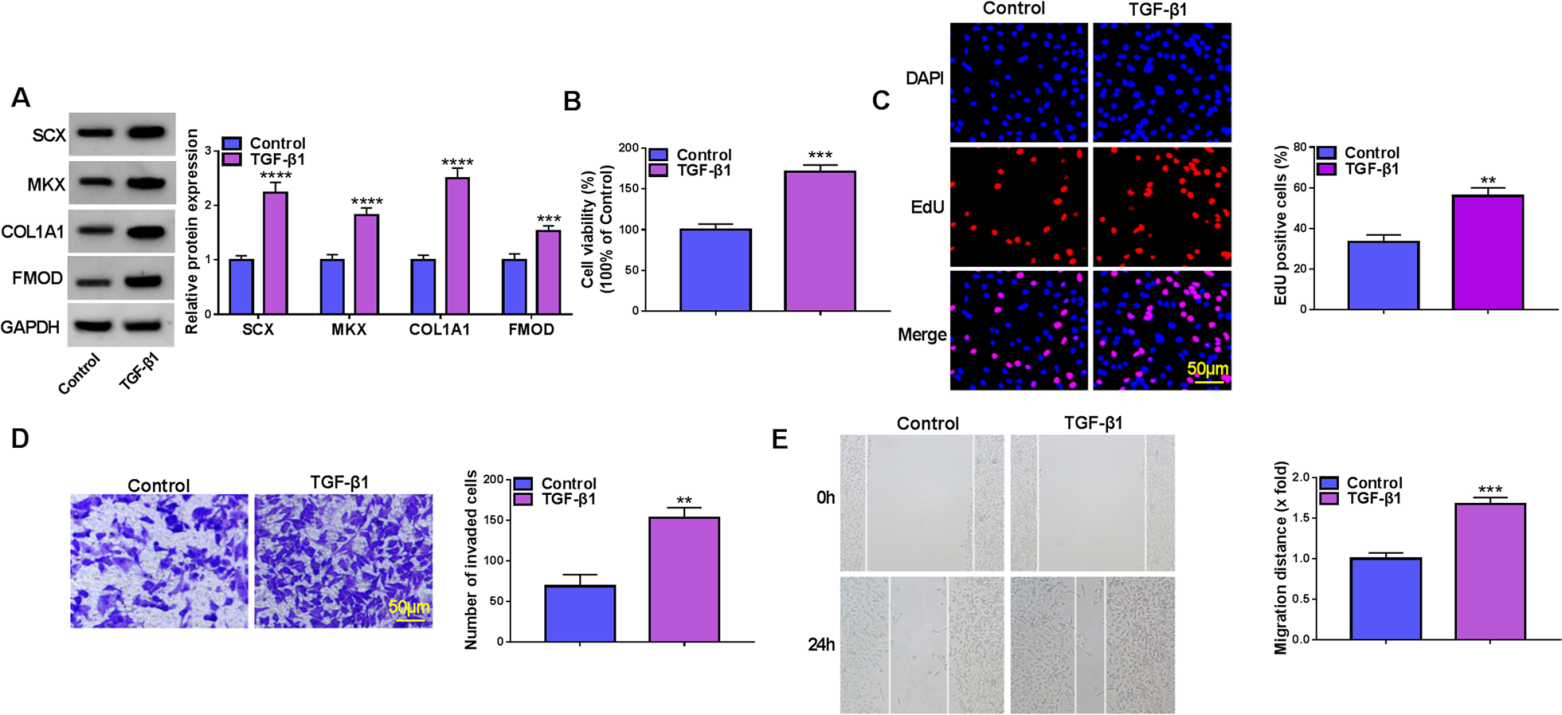
TGF-β1 treatment induces tenogenic differentiation in TDSCs. **A**–**E** TDSCs were treated with 5 ng/mL TGF-β1 for 8 d. **A** Western blotting analysis for the levels of SCX, MKX, COL1A1 and FMOD, and ImageJ was applied to analyze gray values. **B**, **C** Cell proliferation detection by CCK-8 and EdU assays (scale bar, 50 μm). **D** Transwell assay for cell invasion analysis (scale bar, 50 μm). **E** Wound healing assay for cell migration analysis. **P < 0.01, ***P < 0.001, ****P < 0.001

TDSCs were treated with 5 ng/mL TGF-β1; then, tendon-specific markers (SCX, MKX, COL1A1 and FMOD) were detected. Western blotting analysis showed that TGF-β1 treatment elevated the levels of SCX, MKX, COL1A1, and FMOD in TDSCs (Fig. 1A). Next, CCK-8 and EdU assays exhibited that TGF-β1 promoted cell viability and elevated EdU-positive cells in TDSCs (Fig. 1B, C), indicating the promotion of TDSCs proliferation after TGF-β1 treatment. In the meanwhile, TGF-β1 boosted TDSC invasion and migration (Fig. 1D, E). In all, TGF-β1 induced tenogenic differentiation by enhancing the production of tendon-specific markers and TDSC proliferation, invasion and migration.

### TGF-β1 treatment elevates circ_0005736 expression in TDSCs

**Fig. 2:**
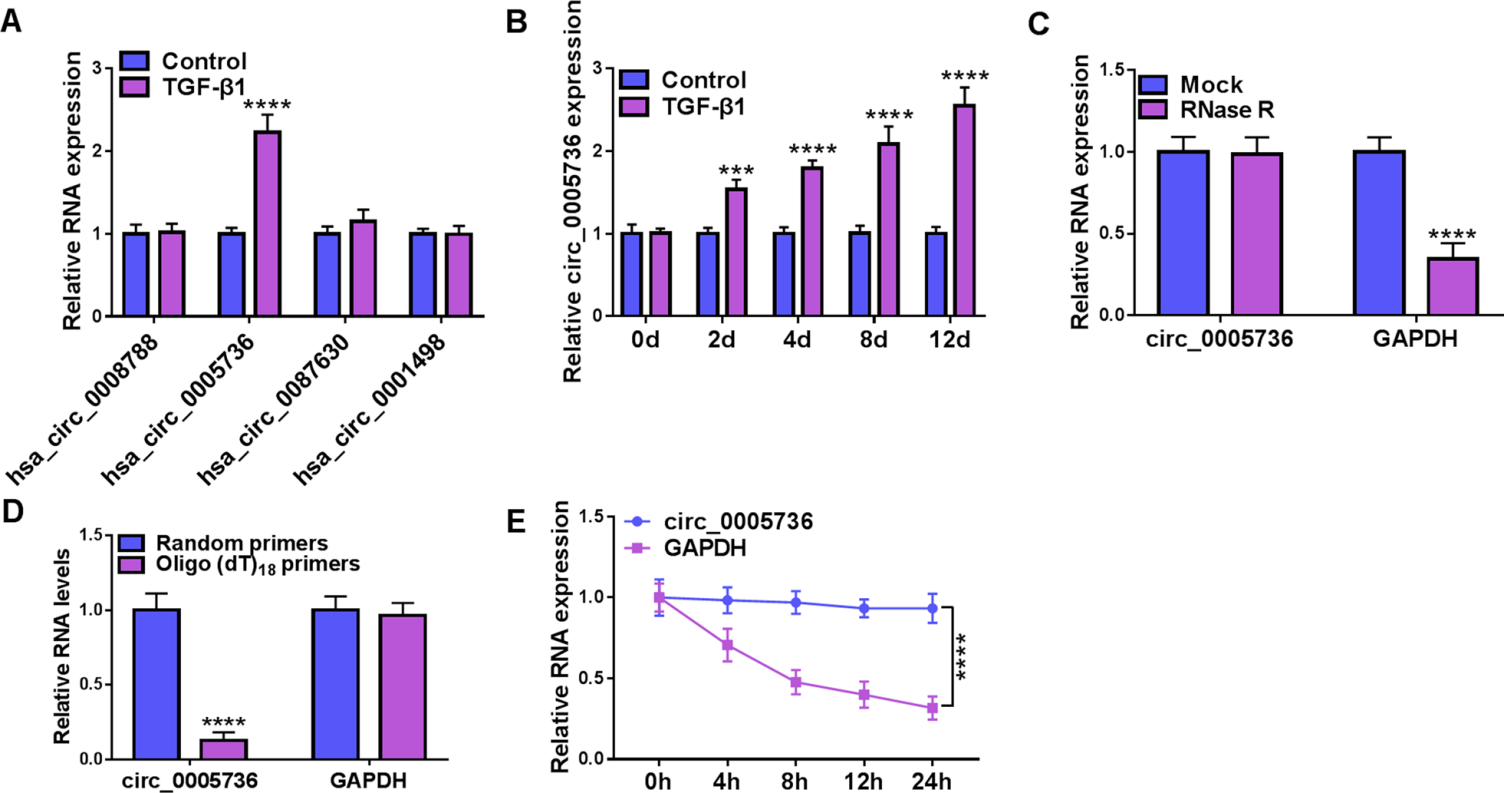
TGF-β1 treatment elevates circ_0005736 expression in TDSCs. **A** qRT-PCR analysis for the levels of hsa_circ_0008788, hsa_circ_0005736, hsa_circ_0087630, and hsa_circ_0001498 in TDSCs treated with TGF-β1. **B** qRT-PCR analysis for circ_0005736 levels in TDSCs treated with TGF-β1 on days 0, 2, 4, 8, and 12. **C** The stability of circ_0005736 was investigated using RNase R treatment in TDSCs. **D** Random and Oligo(dT)18 primers were adopted in reverse transcription to determine the circular structure of circ_0005736. **E** Actinomycin D was used to investigate the stability of circ_0005736 in TDSCs. ***P < 0.001, ****P < 0.001

According to the previous findings, top 4 down-regulated circRNAs in samples of RCT were identified with a cut-off criteria of fold change > 2.0 and *P* < 0.05 [15]. Then, the levels of these 4 circRNAs (hsa_circ_0008788, hsa_circ_0005736, hsa_circ_0087630, hsa_circ_0001498) were detected in TGF-β1-induced TDSCs. As shown in Fig. 2A, hsa_circ_0005736 expression was significantly up-regulated in TDSCs in the presence of TGF-β1. Besides that, we also observed that circ_0005736 expression was increased in TGF-β1-induced TDSCs on days 2, 4, 8 and 12 and reached its highest level on day 12 (Fig. 2B). Thus, circ_0005736 might be involved in TGF-β1 treatment-induced tenogenic differentiation. Then, the characteristics of circ_0005736 were analyzed. Circ_0005736 but not linear GAPDH was resistant to the digestion by RNase R (Fig. 2C). Random and Oligo(dT)18 primers in reverse transcription were used, and we observed circ_0005736 level was lower than linear transcript (Fig. 2D). Besides that, circ_0005736 failed to be digested by Actinomycin D (Fig. 2E), further indicating that circ_0005736 is a stable circular RNA.

### Circ_0005736 silencing reversed TGF-β1-induced tenogenic differentiation in TDSCs

**Fig. 3:**
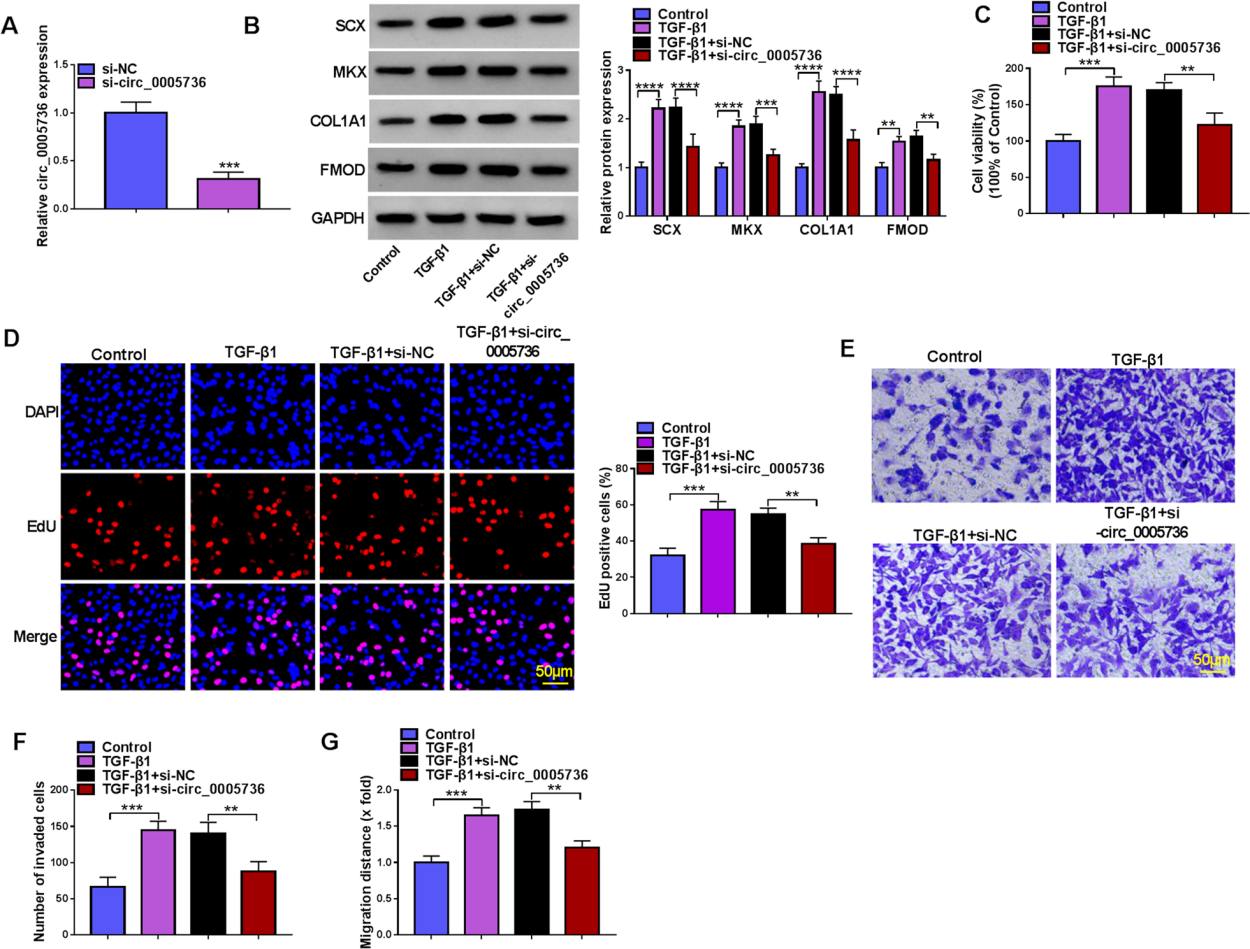
Circ_0005736 silencing reversed TGF-β1-induced tenogenic differentiation in TDSCs. **A** The knockdown efficiency of si-NC and si-circ_0005736 was validated using qRT-PCR. **B**–**G** TDSCs were transfected with si-circ_0005736 or si-NC, followed by TGF-β1 treatment. **B** Western blotting analysis for the levels of SCX, MKX, COL1A1 and FMOD. **C**, **D** Cell proliferation detection by CCK-8 and EdU assays (scale bar, 50 μm). **E**, **F** Transwell assay for cell invasion analysis (scale bar, 50 μm). **G** Wound healing assay for cell migration analysis. **P < 0.01, ***P < 0.001, ****P < 0.001

Next, we explored whether circ_0005736 was involved in TGF-β1-induced tenogenic differentiation in TDSCs. Si-circ_0005736 was designed and transfected into TDSCs, compared with si-NC transfection, si-circ_0005736 introduction markedly decreased circ_0005736 expression in TDSCs (Fig. 3A). Then, transfected cells were treated with TGF-β1 for 8 d. Functionally, circ_0005736 knockdown reversed TGF-β1-mediated increases of the protein of tendon-specific markers (SCX, MKX, COL1A1 and FMOD) (Fig. 3B), enhancement of cell proliferation (Fig. 3C, D), invasion (Fig. 3E, F) and migration (Fig. 3G) in TDSCs.

### MiR-636 is a target of circ_0005736

**Fig. 4:**
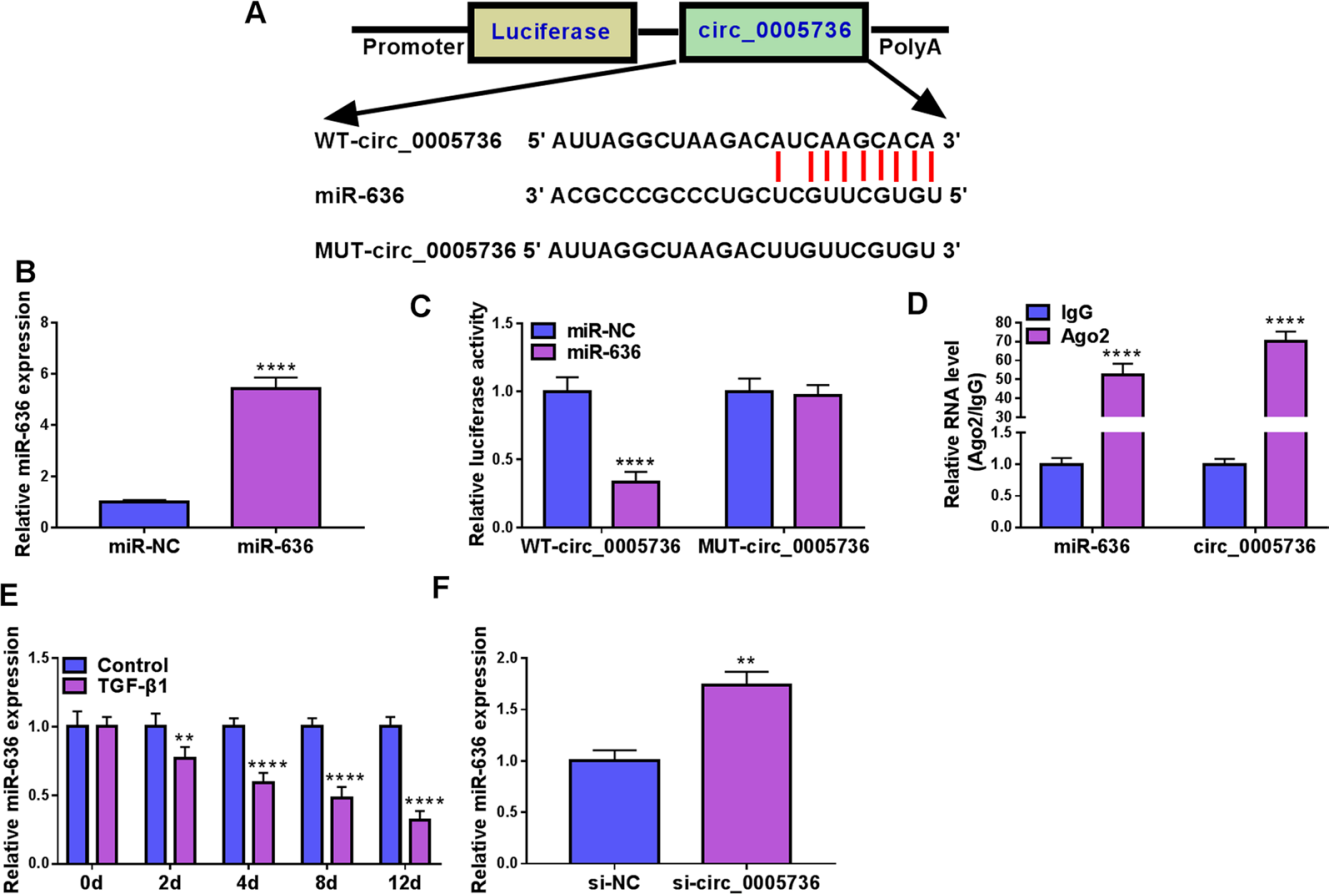
MiR-636 is a target of circ_0005736. **A** The binding sites of miR-636 on circ_0005736. **B** The transfection efficiency of miR-636 mimic or miR-NC was confirmed using qRT-PCR. **C**, **D** Dual-luciferase reporter assay and RIP assay were used for verifying the binding between miR-636 and circ_0005736. **E** qRT-PCR analysis for miR-636 levels in TDSCs treated with TGF-β1 on days 0, 2, 4, 8, and 12. **F** qRT-PCR analysis for miR-636 levels in TDSCs after circ_0005736 knockdown. **P < 0.01, ****P < 0.001

Then, the targets of circ_0005736 were explored. According to the prediction of CircInteractome database, miR-636 has binding sites on circ_0005736 (Fig. 4A). After confirming the transfection efficiency of miR-636 mimic (Fig. 4B), dual-luciferase reporter assay was conducted. The results showed that miR-636 mimic notably reduced the luciferase activity of WT-circ_0005736 group but not MUT-circ_0005736 in TDSCs (Fig. 4C). Further RIP assay showed the enrichment of miR-636 and circ_0005736 in Ago2 antibody compared with the negative IgG antibody (Fig. 4D). MiR-636 expression was found to be decreased in TGF-β1-induced TDSCs on days 2, 4, 8 and 12 (Fig. 4E); moreover, circ_0005736 knockdown led to an increase of miR-636 expression in TDSCs (Fig. 4F). In all, these data confirmed that circ_0005736 directly targeted miR-636.

### Circ_0005736 silencing reversed TGF-β1-induced tenogenic differentiation in TDSCs by miR-636

**Fig. 5:**
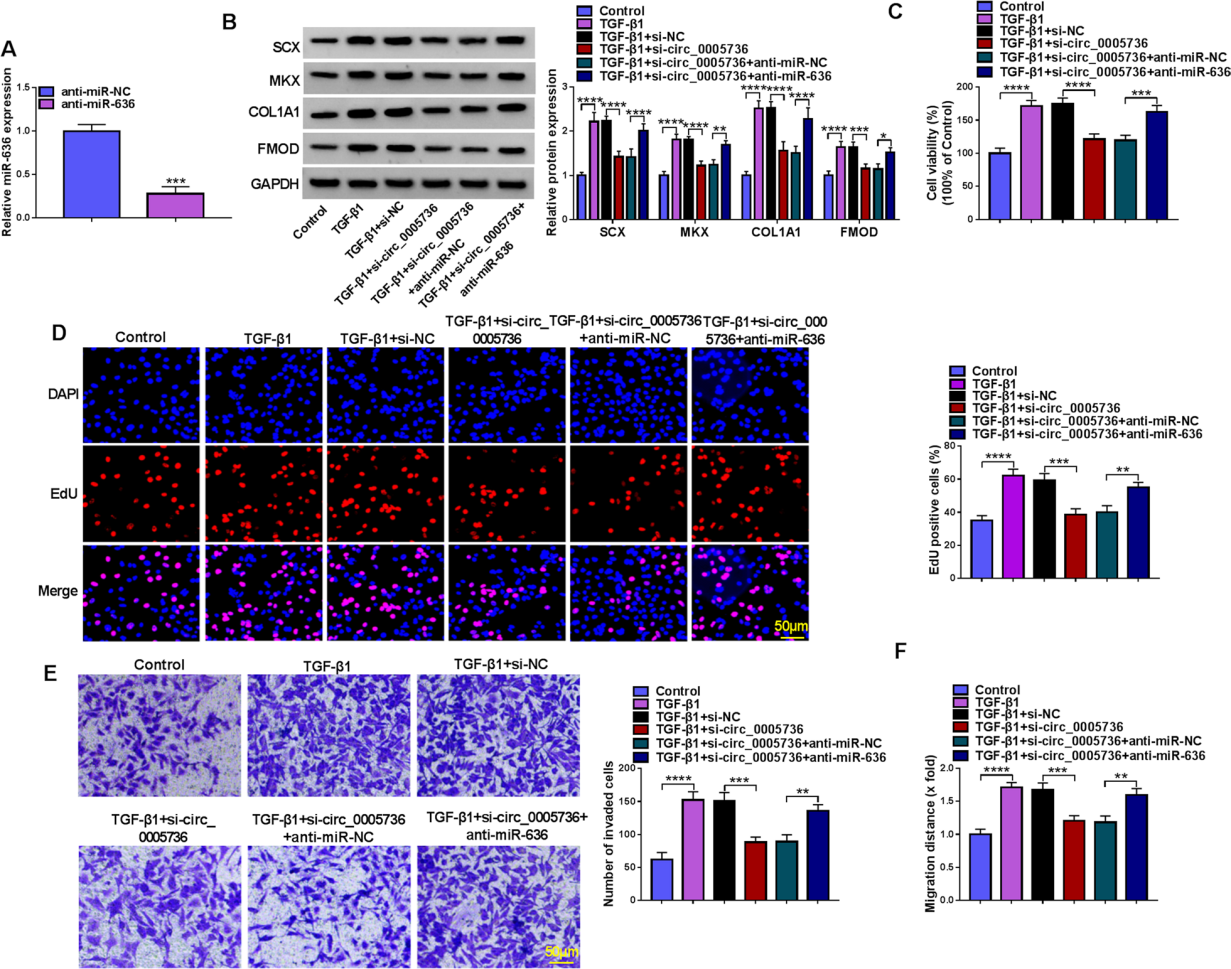
Circ_0005736 silencing reversed TGF-β1-induced tenogenic differentiation in TDSCs by miR-636. **A** The interference efficiency of anti-miR-636 or anti-miR-NC was confirmed by qRT-PCR. **B**–**F** TDSCs were transfected with si-circ_0005736 alone or co-transfected with si-circ_0005736 and anti-miR-636, followed by TGF-β1 treatment. **B** Western blotting analysis for the levels of SCX, MKX, COL1A1 and FMOD. **C**, **D** Cell proliferation detection by CCK-8 and EdU assays (scale bar, 50 μm). **E** Transwell assay for cell invasion analysis (scale bar, 50 μm). **F** Wound healing assay for cell migration analysis. **P < 0.01, ***P < 0.001, ****P < 0.001

The interference efficiency of miR-636 inhibitor (anti-miR-636) was first confirmed. qRT-PCR analysis showed that anti-miR-636 transfection markedly decreased miR-636 expression level in TDSCs relative to anti-miR-NC transfection (Fig. 5A). Then, cells were transfected with si-circ_0005736 alone or co-transfected with si-circ_0005736 and anti-miR-636, followed by TGF-β1 treatment. Functionally, the decreases of the protein levels of tendon-specific markers (SCX, MKX, COL1A1 and FMOD) (Fig. 5B), suppression of cell proliferation (Fig. 5C, D), invasion (Fig. 5E) and migration (Fig. 5F) caused by circ_0005736 deficiency in TGF-β1-induced TDSCs were partly rescued after miR-636 inhibition (Fig. 5B–F). Taken together, circ_0005736 affected TGF-β1-induced tenogenic differentiation in TDSCs via modulating miR-636.

### MAPK1 is a target of miR-636

**Fig. 6:**
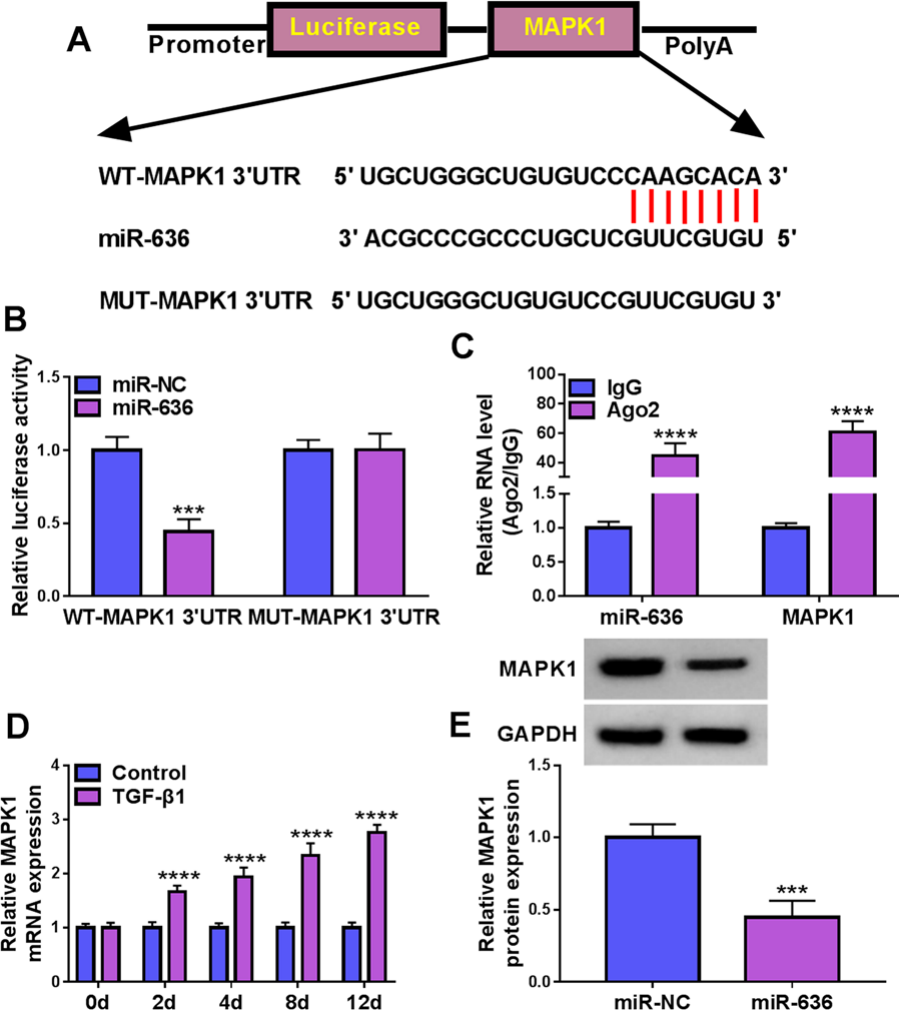
MAPK1 is a target of miR-636. **A** The binding sites of miR-636 on MAPK1. **B**, **C** Dual-luciferase reporter assay and RIP assay were used for verifying the binding between miR-636 and MAPK1. **D** qRT-PCR analysis for MAPK1 levels in TDSCs treated with TGF-β1 on days 0, 2, 4, 8, and 12. **E** Western blotting analysis for MAPK1 protein levels in TDSCs transfected with miR-636 or miR-NC. ***P < 0.001, ****P < 0.001

Subsequently, we explored the targets of miR-636. The database of Targetscan predicted that miR-636 possesses binding sites on MAPK1 (Fig. 6A). Dual-luciferase reporter assay showed that miR-636 overexpression declined the luciferase activity of WT-MAPK1 3’UTR group, but did not affected the luciferase activity of MUT-MAPK1 3’UTR group in TDSCs (Fig. 6B). Then, the enrichment of miR-636 and MAPK1 in Ago2 antibody was verified by RIP assay (Fig. 6C). Thereafter, qRT-PCR exhibited that MAPK1 level was increased in TGF-β1-induced TDSCs on days 2, 4, 8 and 12 (Fig. 6D), and miR-636 mimic reduced MAPK1 level in TDSCs (Fig. 6E). These results confirmed MAPK1 was a target of miR-636.

### MiR-636 overexpression reversed TGF-β1-induced tenogenic differentiation in TDSCs by MAPK1

**Fig. 7:**
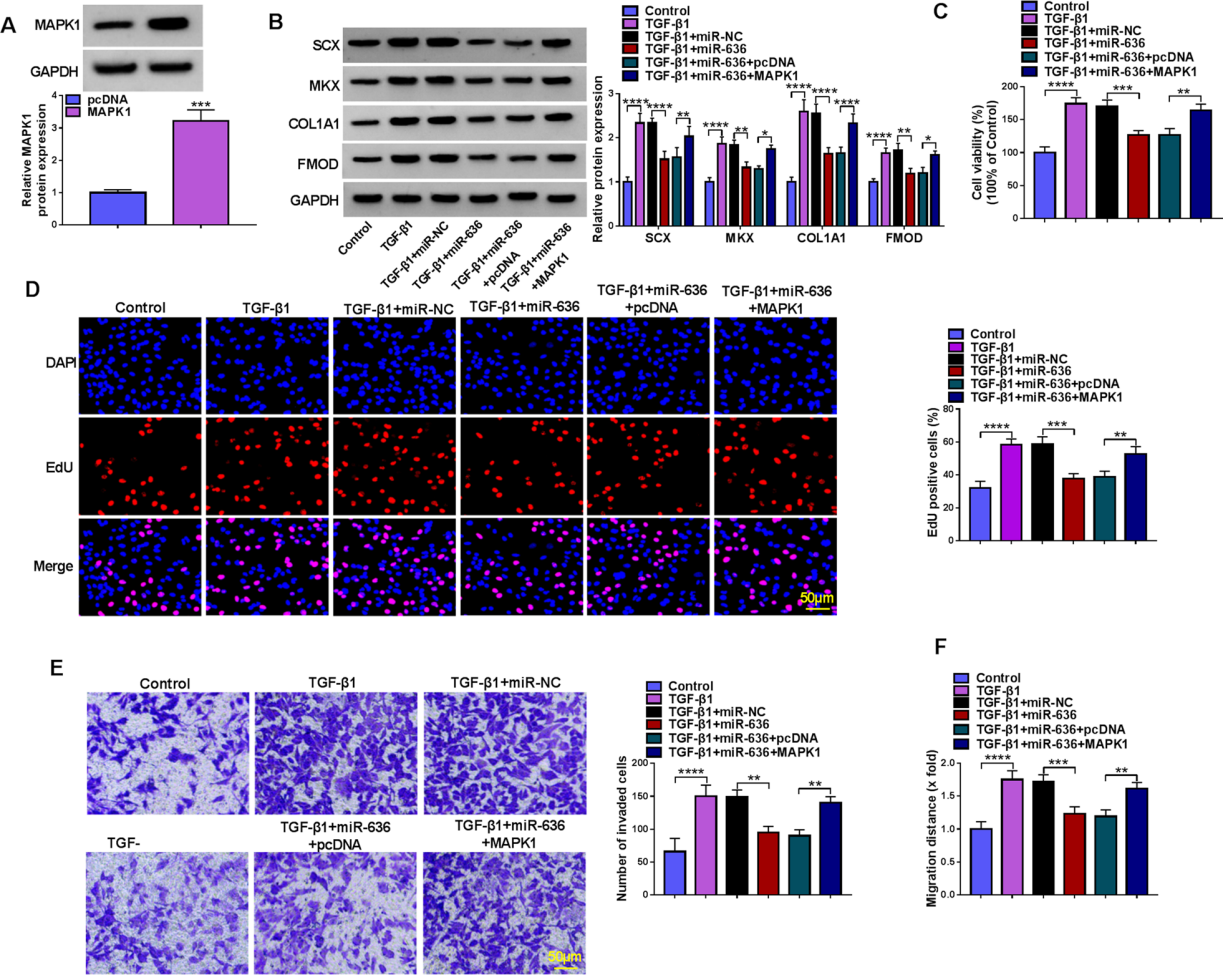
MiR-636 overexpression reversed TGF-β1-induced tenogenic differentiation in TDSCs by MAPK1. **A** Western blotting was adopted to verify the transfection efficiency of pcDNA or MAPK1. **B**–**F** MiR-636 alone or miR-636 and MAPK1 were transfected into TDSCs, and then subjected to TGF-β1 treatment. **B** Western blotting analysis for the levels of SCX, MKX, COL1A1 and FMOD. **C**, **D** Cell proliferation detection by CCK-8 and EdU assays (scale bar, 50 μm). **E** Transwell assay for cell invasion analysis (scale bar, 50 μm). **F** Wound healing assay for cell migration analysis. **P < 0.01, ***P < 0.001, ****P < 0.001

The action of miR-636/MAPK1 axis was then investigated. MAPK1 overexpression plasmids were established, western blotting analysis showed that MAPK1 expression was markedly up-regulated after MAPK1 plasmids introduction (Fig. 7A). Then miR-636 alone or miR-636 and MAPK1 were transfected into TDSCs, and then subjected to TGF-β1 treatment. Functionally, miR-636 up-regulation counteracted TGF-β1-evoked increases of the protein levels of tendon-specific markers (SCX, MKX, COL1A1 and FMOD) (Fig. 7B), promotion of cell proliferation (Fig. 7C, D), invasion (Fig. 7E) and migration (Fig. 7F) in TDSCs, whereas, the effects mediated by miR-636 in TGF-β1-induced TDSCs were reversed by MAPK1 overexpression (Fig. 7B–F). Altogether, miR-636 impacted TGF-β1-induced tenogenic differentiation in TDSCs through MAPK1.

### Circ_0005736/miR-636/MAPK1 forms an axis in TDSCs

**Fig. 8:**
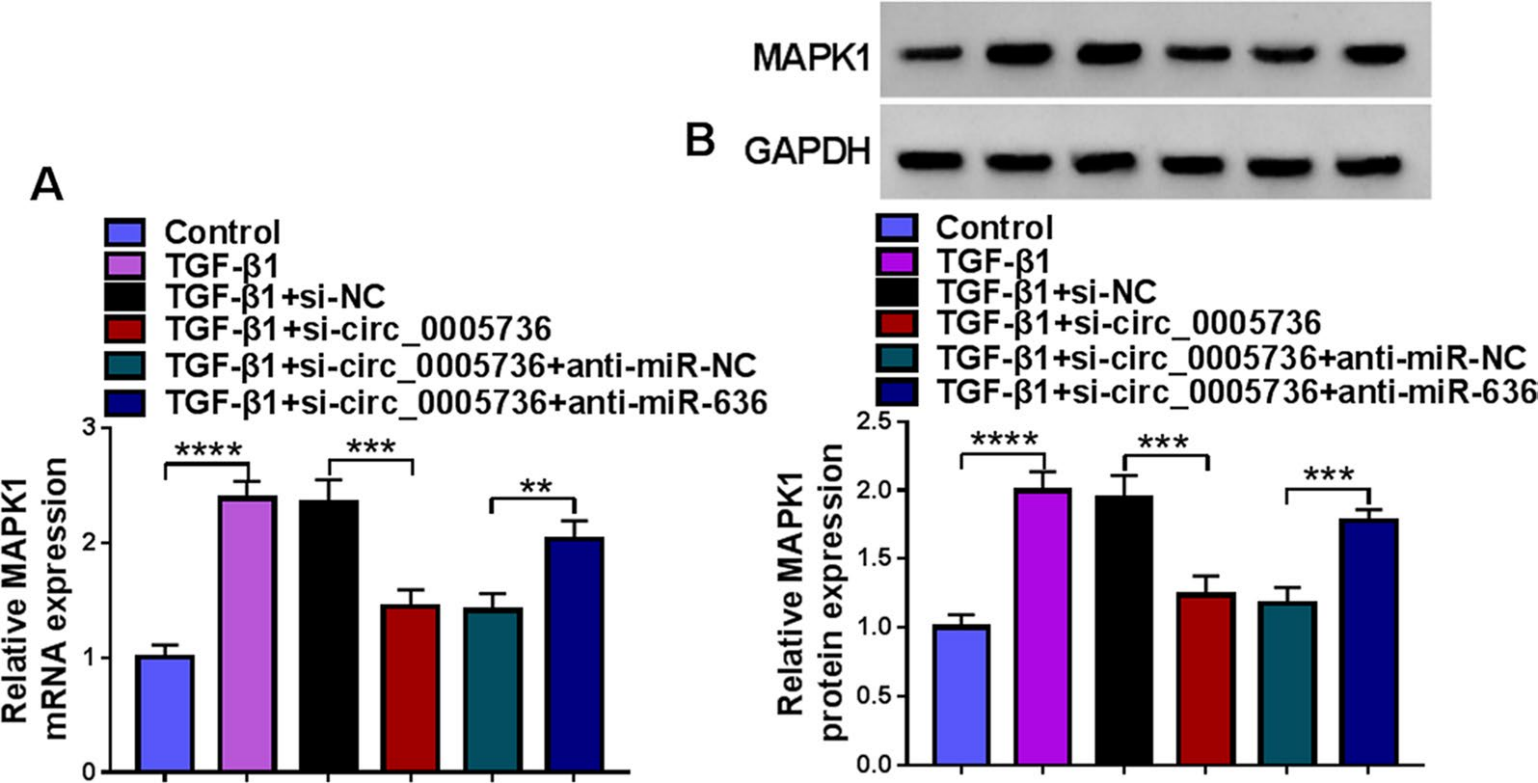
Circ_0005736/miR-636/MAPK1 forms an axis in TDSCs. **A**, **B** TDSCs were transfected with si-circ_0005736 alone or co-transfected with si-circ_0005736 and anti-miR-636, followed by TGF-β1 treatment, and levels of MAPK1 were detected by qRT-PCR and western blotting. **P < 0.01, ***P < 0.001, ****P < 0.001

As displayed in Fig. 8A, B, we found that circ_0005736 deficiency was accompanied with the decreased MAPK1 level in TDSCs under TGF-β1 treatment, while the decrease of MAPK1 mediated by si-circ_0005736 was then rescued by miR-636 silencing, suggesting the circ_0005736/miR-636/MAPK1 axis in TDSCs.

## Discussion

Rotator cuff has a high incidence of tendon injuries, which usually lead to scar tissues with poor mechanical properties and biochemical structures [18]. The regenerative capacity of injured tendons is limited because of their hypocellularity and hypovascularity [19–21]. Currently, growing proof hints at a significant role for cell-based therapies in the repair of tendon injuries [22, 23]. In addition, stem cell-based tissue engineering approaches have also been proposed. Mesenchymal stem cells not only can differentiate in tendon cells, but also secrete several cytokines that regulate inflammation, thereby promoting teno-regenerative events [24]. Human embryonic stem cells (hESC) could be induced to directly differentiate into tendon-like cells by bone morphogenetic protein (BMP)12/13 in the presence of ascorbic acid, enhancing a regenerative tissue healing [25]. TDSCs are a new type of stem cells with the capacities of self-renewal, clonogenicity and multilineage differentiation [26] and are considered to be an ideal cell type for tendon regeneration [27]. Proliferation and mobility are indispensable processes in tendon injuries repair [28]. In this study, we used TGF-β1 to induce tenogenic differentiation in TDSCs, as expected, TGF-β1 treatment evoked tenogenic differentiation in TDSCs by enhancing the production of tenocyte-specific transcription factors (SCX, MKX, and FMOD) and ECM (COL1A1), and triggering TDSC proliferation, invasion and migration. Thereafter, it was found that the levels of circ_0005736 were increased after TGF-β1 treatment, functionally, down-regulation of circ_0005736 reversed the TGF-β1-evoked tenogenic differentiation in TDSCs, indicating the therapeutic effect of circ_0005736 combined with TDSCs in RCT.

Subsequently, the ceRNAs network was identified. This study firstly identified the circ_0005736/miR-636/MAPK1 axis in TDSCs. MiRNAs have been verified to have regulatory functions and may function as therapeutic target for musculoskeletal diseases, including rheumatoid arthritis, osteoarthritis and tendon injuries [29–31]. Yao et al*.* showed the delivery of miR-29a-3p by Exos from umbilical cord stem cells promoted tendon healing [32]. In addition, TDSC-Exos accelerated proliferation, migration and tenogenic differentiation in tenocytes to promote tenon repair via miR-144-3p [33]. Besides that, overexpression of miR-337-3p induced the differentiation of TDSCs, which then attenuated ectopic ossification in tendinopathy rat model [34]. All the data suggested the role of miRNAs and TDSCs in tendon healing. In this work, we found that miR-636 expression was decreased by TGF-β1 treatment, up-regulation of miR-636 abolished TGF-β1 treatment-evoked tenogenic differentiation in TDSCs, moreover, the inhibition of miR-636 could rescue the suppressing action of circ_0005736 knockdown on TDSC tenogenic differentiation caused by TGF-β1, indicating that circ_0005736 affected TDSC tenogenic differentiation by miR-636. MAPKs, also known as ERKs, are key signaling hub to regulate cell differentiation, proliferation, senescence and apoptosis [35]. TDSCs-derived Exo boosted the migration and proliferation of tenocytes by activating MAPK/ERK1/2 and PI3K/AKT pathways, thereby enhancing the healing of injured tendon [36]. The activation of MAPK pathways was involved in the dysfunctions of TDSCs to affect the pathological process of tendinopathy [37, 38]. In our study, we found TGF-β1 treatment promoted the expression of MAPK1, moreover, the suppressing effects of miR-636 on TDSCs tenogenic differentiation was weakened by MAPK1 overexpression.

In all, circ_0005736 enhanced TGF-β1-induced tenogenic differentiation by miR-636/MAPK1 axis. However, the data presented are based on a limited number of cells in vitro. Together with that, the in vivo assay using animal models with high or low circ_0005736 expression is essential to verify these conclusions. Even so, this research also fills the gap in knowledge for the contribution of circ_0005736 in the pathogenesis of RCT, and providing a drug target for therapeutic approach in RCT.

## Data Availability

The data sets used and/or analyzed during the current study are available from the corresponding author on reasonable request.
